# Novel α-MSH analog causes weight loss in obese rats and minipigs and improves insulin sensitivity

**DOI:** 10.1530/JOE-13-0284

**Published:** 2014-01

**Authors:** Keld Fosgerau, Kirsten Raun, Cecilia Nilsson, Kirsten Dahl, Birgitte S Wulff

**Affiliations:** 1 Novo Nordisk Diabetes Research UnitNovo Nordisk A/SNovo Nordisk ParkDK-2760, MaaloevDenmark

**Keywords:** obesity, insulin resistance, hyperinsulinemic–euglycemic clamp, melanocortin receptor 4, agonist, ISI

## Abstract

Obesity is a major burden to people and to health care systems around the world. The aim of the study was to characterize the effect of a novel selective α-MSH analog on obesity and insulin sensitivity. The subchronic effects of the selective MC4-R peptide agonist MC4-NN1-0182 were investigated in diet-induced obese (DIO) rats and DIO minipigs by assessing the effects on food intake, energy consumption, and body weight. The acute effect of MC4-NN1-0182 on insulin sensitivity was assessed by a euglycemic–hyperinsulinemic clamp study in normal rats. Three weeks of treatment of DIO rats with MC4-NN1-0182 caused a decrease in food intake and a significant decrease in body weight 7±1%, *P*<0.05 compared with 3±1% increase with the vehicle control. In DIO minipigs, 8 weeks of treatment with MC4-NN1-0182 resulted in a body weight loss of 13.3±2.5 kg (13±3%), whereas the vehicle control group had gained 3.7±1.4 kg (4±1%). Finally, clamp studies in normal rats showed that acute treatment with MC4-NN1-0182 caused a significant increase in glucose disposal (Rd) compared with vehicle control (Rd, mg/kg per min, 17.0±0.7 vs 13.9±0.6, *P*<0.01). We demonstrate that treatment of DIO rats or minipigs with a selective MC4-R peptide agonist causes weight loss. Moreover, we have demonstrated weight-independent effects on insulin sensitivity. Our observations identify MC4 agonism as a viable target for the treatment of obesity and insulin resistance.

## Introduction

α-Melanocyte-stimulating hormone (α-MSH) belongs to the melanocortins which are derived from a large precursor protein, pre-proopiomelanocortin (pre-POMC). α-MSH has been implicated not only in various behavioral and physiological responses such as pigmentation (Lerner & McGuire 1961), sexual behavior (Bertolini *et al*. 1968), thermoregulation, and inflammatory responses, but also feeding (Vergoni *et al*. 1986) and control of body weight (Fan *et al*. 1997, MacNeil *et al*. 2002). The effect of the melanocortins is mediated through a subfamily of G-protein-coupled receptors with five receptor subtypes denoted MC1-R, MC2-R, MC3-R, MC4-R, and MC5-R, which interact with melanocortin peptides with individual potencies and selectivities (reviewed in Wikberg *et al*. (2000)).

*In vivo* studies on rodents and data from MC4-R-deficient mice, rats, and humans emphasize the importance of MC4-R in body weight regulation. Accordingly, it has been shown in rodents that administration of MC4-R agonists leads to a suppression of appetite and increases the metabolic rate resulting in a significant weight loss (Li *et al*. 2004). Furthermore mice, rats, and humans deficient in the MC4-R are obese (Huszar *et al*. 1997, Vaisse *et al*. 1998, Yeo *et al*. 1998, Mul *et al*. 2012), and data from studies on MC4-R knockout mice show that MC4-R is essential for the mediation of the effect of melanocortins on energy homeostasis; i.e. the presence of MC4-R is necessary for the effects of nonselective melanocortin agonists (melanotan II (MT-II) and BIM22511) on food intake (Marsh *et al*. 1999, Chen *et al*. 2000) and body weight (Kumar *et al*. 2009).

The melanocortin system has been shown to be implicated in acute effects on insulin secretion and/or glucose homeostasis in lean mice (Heijboer *et al*. 2005) as well as in obese mice (Zhou *et al*. 2007, Kumar *et al*. 2009). Also an improvement of glucose tolerance after long-term treatment with melanocortins has been observed in rodents (Banno *et al*. 2004) and in rhesus monkeys (Kievit *et al*. 2013). Interestingly both weight-dependent (Li *et al*. 2005) and weight-independent effects have been described (Obici *et al*. 2001, Lee *et al*. 2007). In contrast, decreased insulin secretion and decreased glucose tolerance after i.c.v. injection of the nonselective melanocortin agonist MT-II into mice was observed in another study (Fan *et al*. 2000). In conclusion, the melanocortin system is considered to be an important player in the central regulation of energy balance and possibly also in the regulation of glucose metabolism.

Although the involvement of the MC4-R in body weight regulation was demonstrated 15 years ago, and many pharmaceutical companies have been engaged in identification of small-molecule MC4-R agonist discovery, no small-molecule drug development program has progressed past phase I, showing that obtaining effective, orally available, and selective small molecule compounds targeting the MC4-R is a difficult task (Sebhat *et al*. 2002, Ujjainwalla & Sebhat 2007). In addition, it has also proved difficult to avoid unwanted side effects such as increased blood pressure and penile erection (Emmerson *et al*. 2007). However, with the increased focus on obesity and the insulin-resistant state of prediabetes, the search for a long-acting MC4-R-selective peptide agonist for s.c. administration may represent an attractive alternative to a small-molecule compound for the treatment of obesity and improvement of insulin sensitivity. As the melanocortin receptors are involved in several different physiological responses, it is important to obtain agonists that are selective for the MC4-R in order to avoid possible side effects derived from activation of the other MC receptors. We have developed a MC4 receptor-selective peptide agonist (binding properties; *K*_i_ values on the respective MC1, MC3, MC4, and MC5 receptors were found to be 400, 42, 0.17, and 10 nM respectively, peptide 11 in Conde-Frieboes *et al*. (2012)) and here the effects of this analog, MC4-NN1-0182, on body weight in obese rats and pigs as well as on glucose utilization in rats are described.

## Materials and methods

The animal experiments were approved by the Danish animal ethics committee. Unless otherwise stated, animals were housed under constant humidity in a temperature (20±2 °C) and light-controlled environment (12 h light:12 h darkness cycle; lights on from 0600 h) with free access to food and water. Rodents had 1–2 weeks of acclimatization before initiation of the studies and the pigs were acclimatized for several months with feed available *ad libitum* before the study was initiated. The α-MSH analog MC4-NN1-0182 was developed at Novo Nordisk A/S as described previously (Conde-Frieboes *et al*. 2012). All chemicals used in the studies were bought from Sigma–Aldrich http://www.sigmaaldrich.com.

### Study 1: subchronic effects of MC4-NN1-0182 in diet-induced obese rats

#### Animals

Male Sprague–Dawley rats (Taconic MB, Ry, Denmark) aged 7–8 weeks were put on a chow control diet (12450B, group A), or a high-fat diet (HFD; 12492, group B-E, Research Diets, New Brunswick, NJ, USA) for 10 weeks. Two weeks before treatment was started the rats were single-housed and acclimatized to injection and handling and then stratified into the following five groups based on body weight (*n*=10): (A) chow control (vehicle); (B) vehicle; (C) MC4-NN1-0182 (0.3 mg/kg); (D) MC4-NN1-0182 (0.1 mg/kg); and (E) pair-fed to C (vehicle). Treatment was given at 1700 h once daily as a single s.c. injection (0.5 ml/kg) for 23 days. The vehicle consisted of sodium chloride (100 mM) and 5% (w/v) hydroxypropylcyclodextrin at pH 5.0. Food intake and body weight were measured daily. Group E was pair-fed to group C, by supplying these animals with the average amount of HFD that was ingested by group C on the previous day.

#### Indirect calorimetry and locomotor activity

Following 15 days of treatment oxygen consumption, respiratory exchange ratio (RER), and locomotor activity were measured in groups B, C, and E using an Oxymax equal flow system (Columbus Instruments, Columbus, OH, USA) equipped with a grid of infrared beams 3.5 cm above the floor level for recording of two-dimensional locomotor activity (Automatik Partner, Glostrup, Denmark). Briefly, the fed animals were placed in the calibrated Oxymax chambers at 1500 h and allowed a 2 h adaptation period before treatment and measurements of O_2_ and CO_2_ concentrations were continued for 21 h. Oxygen consumption was calculated per rat, while RER was calculated as the ratio of CO_2_ production to O_2_ consumption.

#### Oral glucose tolerance test

Following 20 days of treatment, an oral glucose tolerance test (OGTT) was performed as described previously (Madsen *et al*. 2010).

#### Body composition

Total body fat content was analyzed in conscious fed rats using a noninvasive MR scanner (EchoMRI 2004, Echo Medical Systems, Houston, TX, USA) before treatment and on the day before termination.

#### Termination

A blood sample was obtained from isoflurane anaesthetized rats for the determination of plasma glucose, insulin, leptin, and lipids. Leptin was measured using a Linco-plex kit (Linco Research, Inc., St Charles, MO, USA). Plasma levels of nonesterified fatty acid (NEFA), triacylglycerol (TAG), cholesterol (total, LDL, and HDL) were measured on a Hitachi 912 automatic analyzer (Boehringer Mannheim). The mesenteric fat depot was excised and weighed.

### Study 2: chronic effects of MC4-NN1-0182 in diet-induced obese minipigs

#### Animals

Twelve obese female Göttingen minipigs (Ellegaard Göttingen minipigs ApS, Dalmose, Denmark) aged 48 months with a body weight of ∼100 kg were used. The normal body weight of an adult Göttingen minipig is 35–40 kg. A normal body weight can only be maintained if the pigs are fed very restrictively. If they are allowed *ad libitum* access to standard diet (SDS, Scanbur, Sollentuna, Sweden) they will increase the food intake twofold to threefold compared with the restricted amount and the pigs will become obese within half a year. The minipigs are housed in individual pens equipped with feeding devices directly connected to scales for online registration of food intake (MPIGWin Version 2.0, Novo Nordisk, Måløv, Denmark). Two weeks before the onset of the study, the minipigs were dosed daily with 1 ml saline in order to get them accustomed to injections. The minipigs were stratified into two groups (*n*=6) based on body weight. The pigs were treated by s.c. injections for 58 days (8 weeks) with either vehicle: sodium acetate (5 mM)+glycerol (2.54 v/v %), pH 5.0 or MC4-NN1-0182 given as a loading bolus of 30 mg/pig at day 0 followed by dosing every other day with 10 mg/pig. During the entire study, food intake was monitored daily at 15 min intervals for 23.5 h, while body weight was monitored on a scale twice weekly throughout the study.

#### Indirect calorimetry

Following 56 days of treatment, the minipigs (*n*=3) were placed in a custom built respiration chamber equipped with a feeding system and water supply at 1400 h. Measurement of O_2_ and CO_2_ concentrations was started when the lights were turned off at 1700 h. Lights were turned back on at 0715 h, the following day and the measurements were stopped at 0900 h. Oxygen consumption was calculated as described earlier.

### Study 3: acute effects of MC4-NN1-0182 in hyperinsulinemic–euglycemic clamp in rats

#### Animals

Male Sprague–Dawley rats (Taconic MB) aged 5–6 weeks had permanent catheters implanted in the portal and jugular vein and in the carotid artery followed by a 2-week recovery period as described previously (Fosgerau *et al*. 2006). The day before the clamp experiment, the animals were semi-fasted to 70% of normal food intake overnight. A total of 13 unrestrained and conscious rats were clamped and treated with either vehicle (NaCl, 100 mM; hydroxypropylcyclodextrin, 5% (w/v), *n*=7) or MC4-NN1-0182 (*n*=6).

#### Hyperinsulinemic–euglycemic clamp protocol

The clamp protocol is described by Finegood *et al*. (1987). Blood was drawn from the artery at *t*=−155 min for baseline values of blood glucose, plasma insulin, and glucose-specific activity (GSA). Then, at *t*=−153 min, a s.c. (0.5 ml/kg) injection of either vehicle or MC4-NN1-0182 (1 mg/kg) was given. At *t*=−151 min, constant infusion of ^3^H-3-d-glucose (80 μCi/kg+0.8 μCi/kg per min, Perkin Elmer, Waltham, MA, USA) was initiated. Blood was drawn every 15 min at *t*=−150 to −30 min and every 6 min at *t*=0 to 120 min for immediate measurement of plasma glucose. Samples were obtained every 6 min at *t*=−30 to 0 min and *t*=90 to 120 min for the determination of blood glucose, plasma insulin, and GSA. Plasma glucose was determined using a YSI 2300 autoanalyzer (YSI, Inc., Yellow Springs, OH, USA), and insulin was measured as described previously (Andersen *et al*. 1993). Portal infusion of insulin (20 mU/kg+4.5 mU/kg per min, Actrapid, Novo Nordisk) was initiated at *t*=0 min and continued until the end of the study at *t*=120 min and at the same time a variable-labeled glucose infusion (Ginf, 1 μCi/10 mg) was given in the jugular vein for maintaining euglycemia. All blood samples were collected in tubes containing NaF (12.5 mg/ml) and heparin (30 IE/ml) and plasma were separated by centrifugation. Then, following precipitation with Ba(OH)_2_ and ZnSO_4_ GSA in the protein-free supernatant of plasma was determined by liquid scintillation of radioactivity (Beckman LS 6000 TA, Ramcon, Birkerød, Denmark).

#### Glucose utilization

Endogenous glucose production (EGP) and whole-body glucose uptake (Rd) were measured in two defined steady-state periods (SS1, *t*=−30 to 0 min, normoinsulinemia; SS2, *t*=90 to 120 min, hyperinsulinemia) using conventional tracer technique with the modified Steel's one-compartment model (Finegood *et al*. 1987). To express the ability of insulin to suppress EGP, we set the rate of EGP in the first steady-state period to 100%.

### Statistical analysis

Data are expressed as mean±s.e.m. unless otherwise stated. Statistical evaluations of the data were done using one-way or two-way ANOVA followed by appropriate *post hoc* analysis. All statistical calculations were performed using Prism 5.0 software (GraphPad Software, Inc., San Diego, CA, USA), and *P*<0.05 was considered statistically significant. The index of insulin sensitivity (ISI) was determined as described previously (Matsuda & DeFronzo 1999).

## Results

### Study 1: subchronic effects of MC4-NN1-0182 in diet-induced obese rats

**Figure 1:**
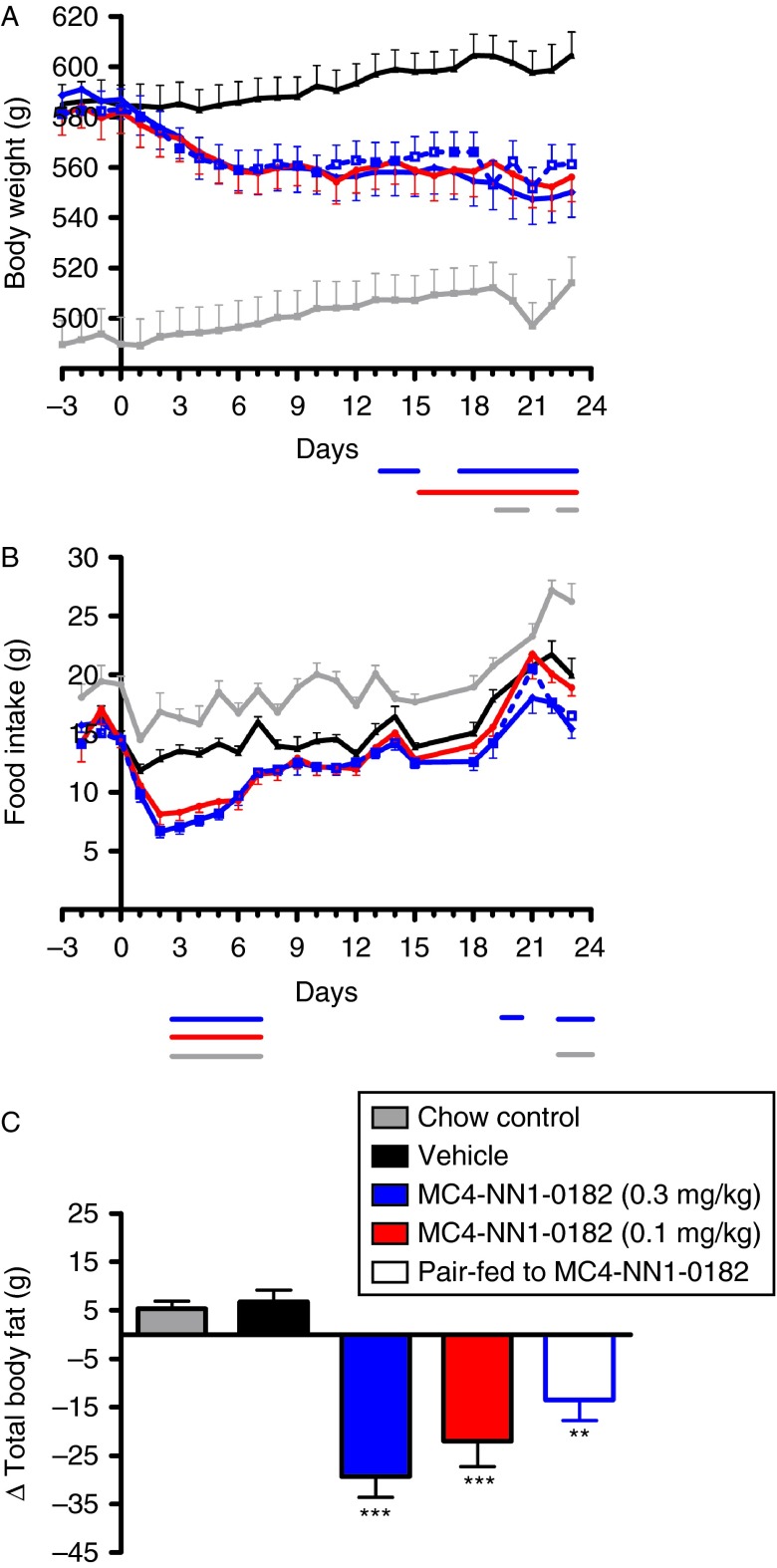
Effect of MC4-NN1-0182 on food intake, body weight, and composition in DIO rats. Effect of s.c. administration of MC4-NN1-0182 (dark blue line/bar, 0.3 mg/kg – red line/bar, 0.1 mg/kg) or vehicle control (black line/bar), chow control (vehicle, grey line/bar), or pair-fed to MC4-NN1-0182 0.3 mg/kg (vehicle, dashed line/open bar) for 23 days on body weight (A), daily food intake (B), and changes in total body fat (C) in DIO rats, see Materials and methods for details. Data are mean±s.e.m., *n*=10/group. Data were compared by two-way ANOVA followed by Bonferroni's *post hoc* test where *P*<0.05 vs vehicle control are indicated by horizontal bars below the x-axis scale, or by one-way ANOVA followed by Bonferroni's multiple comparison test where ***P<0.001, **P<0.01 vs vehicle control.

We observed a dose-dependent decrease in food intake in diet-induced obese (DIO) rats treated with MC4-NN1-0182 as compared with vehicle control (Fig. 1B). This decrease was significant (*P*<0.05) in the first week of dosing and remained below the level for the vehicle control group for the entire study at both doses of MC4-NN1-0182. Further, as compared with vehicle control, administration of MC4-NN1-0182 caused a dose-dependent decrease in body weight (Fig. 1A), which was paralleled by a significant decrease in total body fat and mesenteric fat (Fig. 1C, Table 1, *P*<0.05) and plasma levels of leptin (Table 1). Thus, at study termination, we observed a significant (*P*<0.05) decrease in body weight of 7±1% (*P*<0.05) and 4±1% (*P*<0.05) in DIO rats treated with MC4-NN1-0182 at 0.3 and 0.1 mg/kg respectively. This was compared with a 4±1% decrease in the group pair-fed to the 0.3 mg/kg group and a 3±1% increase for the vehicle control group. The effect of MC4-NN1-0182 on plasma lipids are shown in Table 1. We observed a significant (*P*<0.05) effect on total plasma levels of cholesterol in DIO rats treated with MC4-NN1-0182 as compared with vehicle control, but not on levels of NEFA or TAG (*P*=NS).

**Figure 2:**
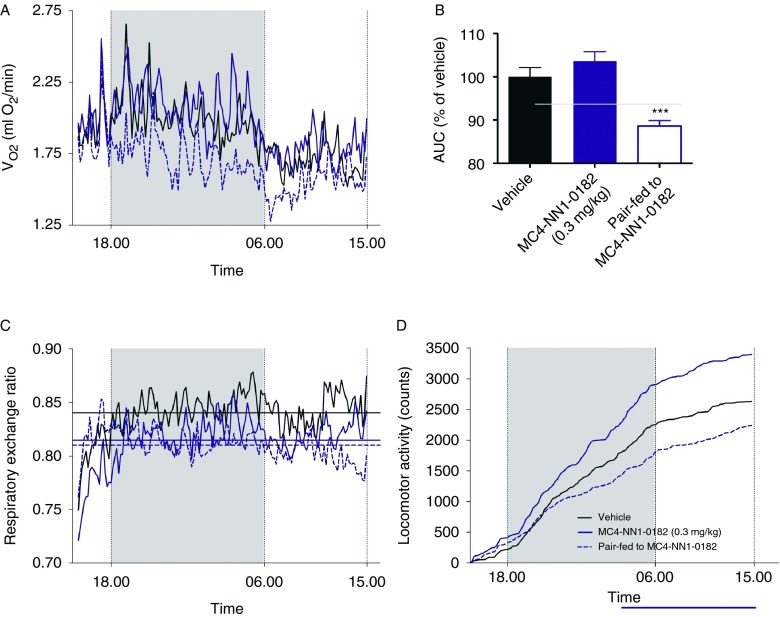
Effect of MC4-NN1-0182 on oxygen consumption, respiratory exchange ratio (RER), and locomotor activity in DIO rats. Effect on 23-h oxygen consumption of s.c. administration of MC4-NN1-0182 (dark blue line/bar, 0.3 mg/kg), vehicle control (black line/bar), or pair-fed to MC4-NN1-0182 0.3 mg/kg (vehicle, dashed line/open bar) following 15 days of treatment (A and B), the RER (C) with averages illustrated by vertical lines, and the 23-h accumulated locomotor activity (D) in DIO rats, see Materials and methods for details. Data are mean±s.e.m., *n*=10 or 5/group. Data were compared by two-way ANOVA followed by Bonferroni's post-test where *P*<0.05 vs vehicle control are indicated by vertical bars, or by one-way ANOVA followed by Bonferroni's multiple comparison test where ***P<0.001 vs vehicle control.

Rats treated with MC4-NN1-0182 (0.3 mg/kg) displayed a similar rate of oxygen consumption (Fig. 2A and B, *P*=NS) to those in the vehicle control group. In contrast, we observed significantly a (*P*<0.001) lower level of oxygen consumption in the pair-fed group as compared with the vehicle control group. Along with the changes in oxygen consumption, we observed a non-significant trend toward a decrease in the RER (Fig. 2C, *P*=NS) in MC4-NN1-0182 and pair-fed animals compared with vehicle control. We observed significantly a higher activity in the animals treated with MC4-NN1-0182 compared with the pair-fed group (*P*<0.05, Fig. 2D). However, no difference was observed when comparing the MC4-NN1-0182 or the pair-fed group with the vehicle control group (*P*=NS).

**Figure 3:**
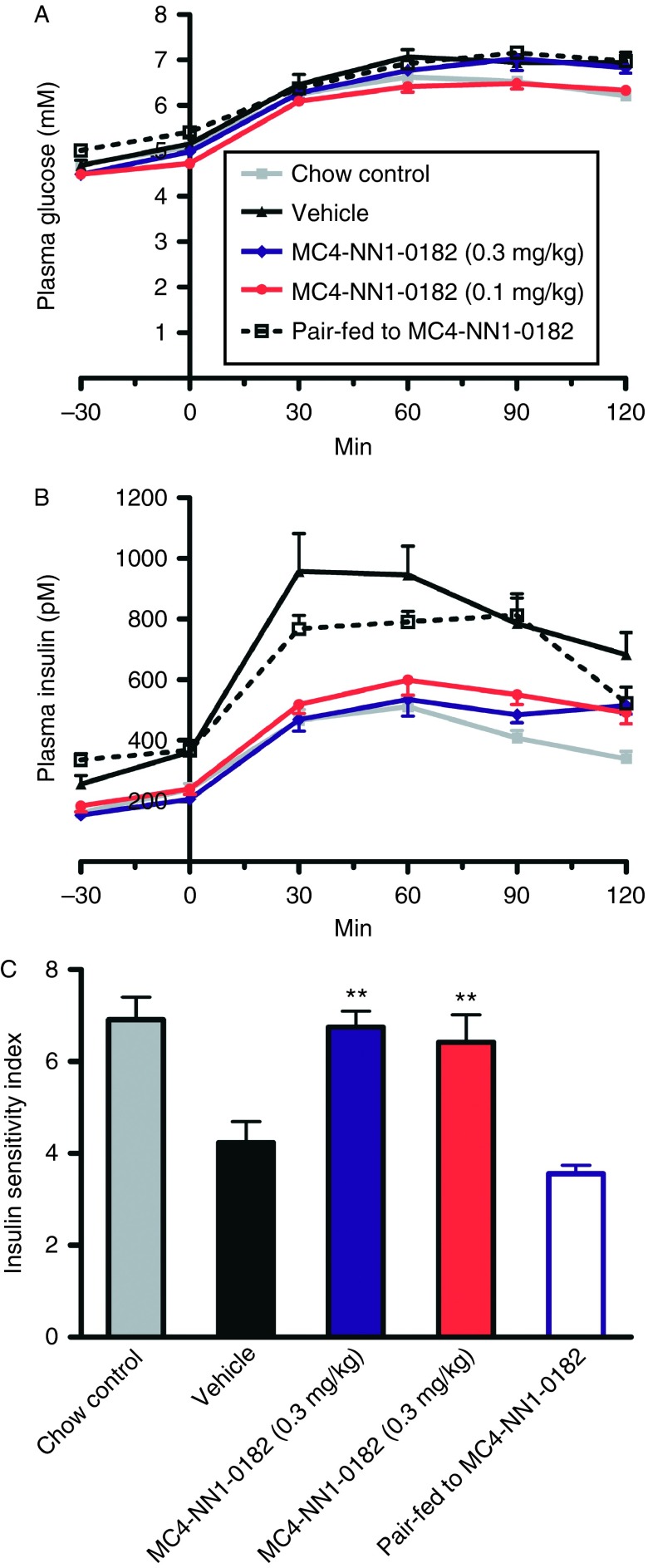
Effect of MC4-NN1-0182 on insulin sensitivity in DIO rats. Effect of s.c. administration of MC4-NN1-0182 (dark blue line/bar, 0.3 mg/kg – red line/bar, 0.1 mg/kg) or vehicle as control (black line/bar), chow control (vehicle, grey line/bar), or pair-fed to MC4-NN1-0182 0.3 mg/kg (vehicle, dashed line/open bar) following 20 days of treatment on the plasma glucose (A) and insulin (B) in rats following an oral glucose tolerance test (OGTT). Also, shown in (C) is the calculated insulin sensitivity index (ISI), see Materials and methods for details. Data are mean±s.e.m., *n*=10/group. Data were compared by one-way ANOVA followed by Bonferroni's multiple comparison test where **P<0.01 vs vehicle control.

Figure 3A, B, and C shows the data from the OGTT. The insulin sensitivity in the vehicle high-fat control group was significantly lower than in vehicle-treated rats fed with a normal chow (*P*<0.01). Treatment with MC4-NN1-0182 restored the insulin sensitivity to the level observed in the chow-control groups (Fig. 3B). Also, a lower plasma level of insulin was observed in DIO rats treated with MC4-NN1-0182 compared with the vehicle control group at the termination of the study endpoint (Table 1).

### Study 2: chronic effects of MC4-NN1-0182 in DIO minipigs

**Figure 4:**
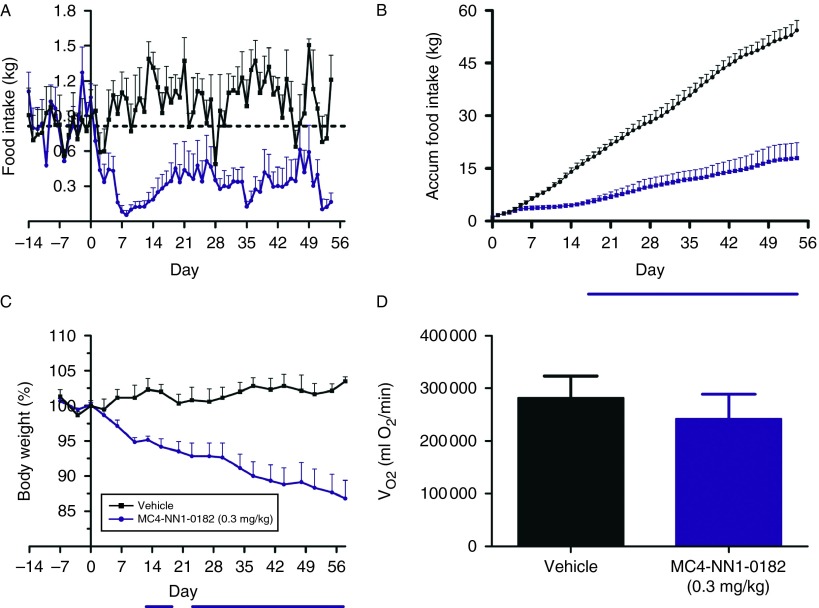
Effect of MC4-NN1-0182 on food intake, body weight, and oxygen consumption in obese minipigs. Effect of s.c. administration of MC4-NN1-0182 (dark blue line/bar, 0.3 mg/kg bolus+0.1 mg/kg every other day) or vehicle control (black line/bar) for 56 days on daily (A) and accumulated (B) food intake, body weight, (C) and the oxygen consumption (D) in obese minipigs. Data are mean±s.e.m., *n*=6/group except for oxygen consumption (D) which is *n*=3/group). Pigs treated with MC4-NN1-0182 were compared with the vehicle control group using a two-way ANOVA followed by Bonferroni's multiple comparison test where *P*<0.05 is indicated by a horizontal blue line below the x-axis scale.

We observed an immediate and significant decrease in food intake in DIO minipigs dosed with MC4-NN1-0182 compared with vehicle control (Fig. 4A, *P*<0.05). The decrease in food intake was sustained throughout the study. A significant reduction in body weight could be observed following 9 days of treatment and for the rest of the entire study (Fig. 4C, *P*<0.05). At endpoint pigs treated with MC4-NN1-0182 had lost 13.3±2.5 kg (13±3%), whereas the vehicle control group had gained 3.7±1.4 kg (4±1%). VO_2_ (ml O_2_/min) was 281 691±41 898 for the vehicle group vs 241 895±41 898 for the MC4-NN1-0182-treated group (Fig. 4D). No difference was observed in the oxygen consumption measurement after 8 weeks of treatment when comparing the two groups (*P*=NS).

### Study 3: acute effects of MC4-NN1-0182 in hyperinsulinemic–euglycemic clamp in rats

**Figure 5:**
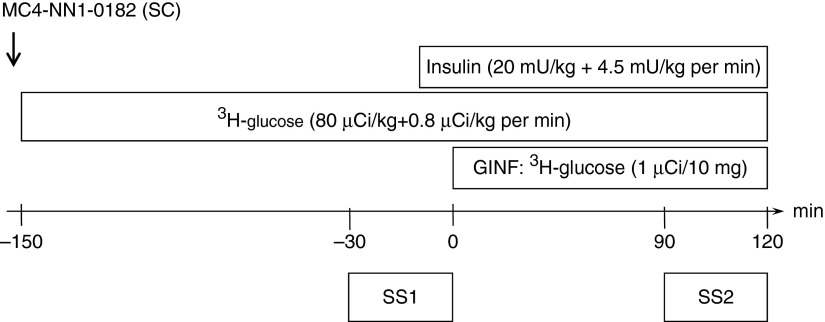
Protocol for injections and infusions in clamp study in normal rats. Blood was drawn from the artery at *t*=−155 min for baseline values of blood glucose, plasma insulin, and glucose specific activity (GSA). Then, at *t*=−153 min a s.c. injection of either vehicle or MC4-NN1-0182 (1 mg/kg) was given. At *t*=−151 min constant infusion of ^3^H-3-d-glucose was initiated. Portal infusion of insulin was initiated *t*=0 min and continued until the end of the study at *t*=120 min and at the same time a variable hot glucose infusion (Ginf, 1 μCi/10 mg) was given in the jugular vein for maintaining euglycemia. Glucose utilization: endogenous glucose production (EGP) and whole-body glucose uptake (Rd) were measured during two steady-state periods. The first steady-state period (SS1, *t*=−30 to 0 min) was under basal conditions and normoinsulinemic–euglycemic conditions. The second steady-state period was defined as stable hyperinsulinemic–euglycemic conditions during the last 30 min of the clamp (SS2, *t*=90 to 120 min).

**Figure 6:**
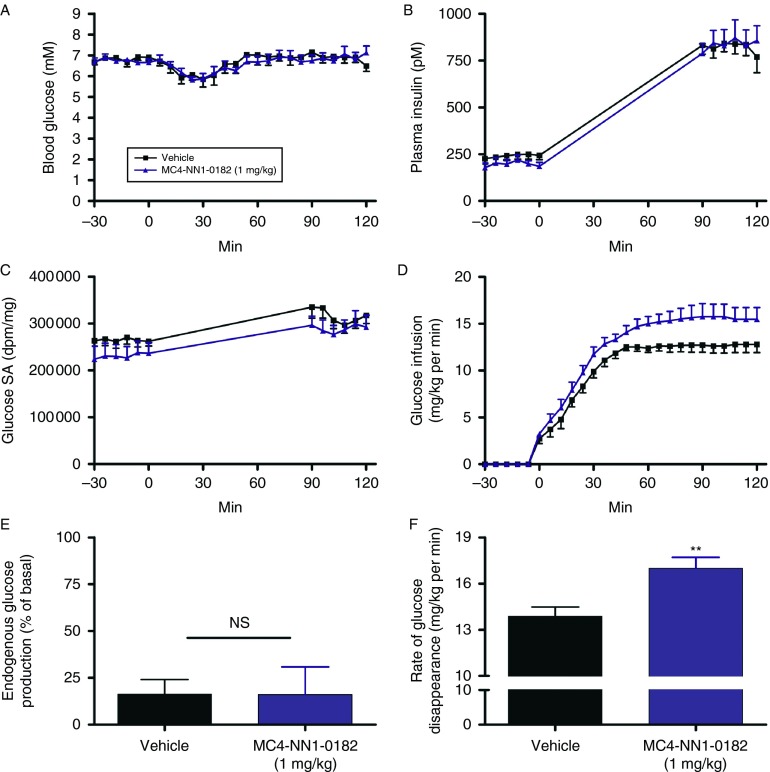
Acute effect of MC4-NN1-0182 on glucose utilization sensitivity in normal rats. Effects of a single acute s.c. injection of MC4-NN1-0182 (1.0 mg/kg (dark blue line/bar) or vehicle control (black line/bar) in normal rats during a hyperinsulinemic–euglycemic clamp study are shown. Measured levels of plasma glucose (A), insulin (B), and glucose-specific activity (GSA, C) are given. Also, the glucose infusion rate (Ginf) necessary to maintain euglycemia is shown (D). Finally, calculated suppression of endogenous glucose production (EGP, E) and rate of glucose disappearance (Rd, F) are shown during the hyperinsulinemic period at the end of the clamp (*t*=90–120 min), see Materials and methods for details. Data are mean±s.e.m., *n*=6/group. Rats treated with MC4-NN1-0182 were compared with vehicle control using an unpaired Student's *t*-test where ** indicates *P*<0.01 vs vehicle control.

An overview of the clamp protocol is represented in Fig. 5 and is described by Finegood *et al*. (1987). By infusion of exogenous glucose, the plasma glucose level was maintained at euglycemia during the entire clamp from *t*=−30 to 120 min and we observed no differences between the two groups (Fig. 6A, *P*=NS). The plasma levels of insulin (Fig. 6B) were raised about threefold in both groups from the first steady-state period (SS1) to the second (SS2). Finally, we observed no differences in the GSA in the two groups during both steady-state periods (Fig. 6C, *P*=NS). Taken together, the clamp conditions enabled a qualified determination of glucose turnover. The glucose infusion necessary to maintain euglycemia is shown in Fig. 6D. Significantly less glucose was used in the animals treated with vehicle control vs MC4-NN1-0182 (AUC, mg/kg, 1303±37 vs 1567±60, *P*<0.01). In the first steady-state period under normoinsulinemia, the basal EGP was similar in the two groups (EGP, mg/kg per min, 6.1±0.3 vs 6.8±0.5 vehicle vs MC4-NN1-0182, *P*=NS) and this was paralleled by equal basal rate of glucose disappearance (Rd, mg/kg per min, 6.2±0.3 vs 6.8±0.5, vehicle vs MC4-NN1-0182, *P*=NS). Figure 6E display the ability of hyperinsulinemia to suppress EGP. We observed no differences between animals treated with MC4-NN1-0182 vs the vehicle control (% of basal EGP, 15±8 vs 16±8, vehicle vs MC4-NN1-0182, *P*=NS). Figure 6F displays the effect of hyperinsulinemia on Rd. We observed a significant increase in Rd in animals treated with MC4-NN1-0182 as compared with vehicle control (Rd, mg/kg per min, 17.0±0.7 vs 13.9±0.6, *P*<0.01).

## Discussion

Obesity and the well-documented comorbidities such as type 2 diabetes, cardiovascular diseases, and cancer are a challenge to the health care systems. Currently body weight normalization in severe obesity is often not reached. Therefore, the search for effective weight lowering drugs remains very relevant as complement to existing therapies (Nguyen *et al*. 2012). Insulin-resistance plays a significant role in both obesity and prediabetes (Reaven 1988, Ferrannini 1993) and the reversal of the insulin-resistant state could prevent the development of several metabolic diseases. The MC4-R has been shown to be involved in the regulation of body weight (Huszar *et al*. 1997, Vaisse *et al*. 1998, Yeo *et al*. 1998, Mul *et al*. 2012), and is recognized as a promising target for therapeutically treating obesity. Here, we report the effects of the MC4-R-selective peptide agonist MC4-NN1-0182 on obesity in DIO rats and DIO minipigs. Moreover, this is the first study, to our knowledge, to address the acute effects of MC4-R agonism on insulin sensitivity by a hyperinsulinemic clamp in normal rats.

Treatment with the selective agonist MC4-NN1-0182 significantly reduced food intake and body weight in both DIO rats and DIO minipigs. In DIO rats, the loss of body weight was paralleled by a loss of mesenteric fat and a decrease in plasma leptin levels, reflecting the expected effects on the fat tissue. The effect on body weight in DIO rats was greater in the treated group than in the pair-fed group, indicating that the agonist stimulates energy expenditure. However, we observed only a trend toward an increase in oxygen consumption in the MC4-NN1-0182 group after 2 weeks of treatment. In contrast, in the pair-fed group, we observed decreased oxygen consumption, probably in an attempt to preserve body weight. Similar finding have been reported for MT-II (Pierroz *et al*. 2002).

In the DIO minipigs, MC4-NN1-0182 treatment resulted in a significant and sustained effect on food intake, resulting in an overall reduction in food intake of 68% after 8 weeks treatment. The body weight difference between the treated and control groups was ∼17 kg after 8 weeks of treatment. As in the DIO rats, no significant difference in oxygen consumption was observed in the DIO minipigs. As food intake in the MC4-NN1-0182-treated DIO rats and minipigs was lower than in the vehicle control groups, and as the sustained decrease in food intake and body weight would be expected to cause a physiological decrease in energy expenditure (Clapham & Arch 2011), the observation that oxygen consumption is in fact similar, is interesting: it can be speculated that the MC4-R agonist MC4-NN1-0182 may be able to counteract the physiologically expected decrease in energy expenditure.

Taken together, the effect on food intake was evident, and an effect on energy expenditure also seemed to be involved in the impressive weight loss effects observed with this long-acting MC4 agonist. The exact mechanism for the proposed effect on energy expenditure in DIO rats is not known, but brown fat is known to be very metabolically active in rodents (Cannon & Nedergaard 2004) and stimulation of this fat compartment could be an obvious explanation for this effect. The finding that the oxygen consumption was not decreased in the minipigs in relation to the large weight loss and thus indicating some degree of effect on energy expenditure is especially interesting as pigs do not express uncoupling protein 1 (UCP1), e.g., pigs do not have any brown fat (Berg *et al*. 2006). We speculate that such an effect on energy expenditure may therefore be generated by increased metabolism in muscles, an effect that could be very relevant and beneficial for obese humans.

We report that acute injection of MC4-NN1-0182 in lean rats has a significant effect on insulin sensitivity as reflected by a 22% increase in peripheral glucose uptake (Rd, Fig. 6F) compared with vehicle control under hyperinsulinemic conditions, while no effect on hepatic insulin sensitivity is reported. These results are consistent with previous results in mice after treatment with the nonselective MT-II (Heijboer *et al*. 2005). In DIO rats, an OGTT was performed and the effect of MC4-NN1-0182 on insulin sensitivity was evaluated by using the calculated ISI. Interestingly, the ISI of MC4-NN1-0182-treated DIO rats was similar to that of chow-control animals, whereas the pair-fed group, which lost body weight similarly to animals in the low-dose MC4-NN1-0182 group, displayed a lower ISI. Similarly, the circulating levels of insulin in MC4-NN1-0182-treated animals, but not in pair-fed control, were lower than those of vehicle-treated DIO rats at the time of termination. Collectively, these data indicate that MC4-NN1-0182 may affect insulin sensitivity by a mechanism independent of the associated body weight loss. These observations are in agreement with previously reported data on insulin sensitivity; i.e. from a comparison of subchronic MT-II-treated rats dosed either centrally or peripherally and compared with pair-fed controls (i.c.v. administration to lean or DIO rats (Obici *et al*. 2001) or s.c. administration to OLETF rats (Banno *et al*. 2004)). The effect on insulin sensitivity is probably, at least in part, centrally mediated as a similar effect was also obtained after i.c.v. injection (Obici *et al*. 2001). Furthermore, Hill *et al*. (2010) showed that direct insulin and leptin actions on POMC neurons are required for normal glucose homeostasis, also indicating central-mediated effects of melanocortins on glucose homeostasis. As our analog is specific for the MC4-R (Conde-Frieboes *et al*. 2012), our data indicate MC4-R to be the main mediator of these effects.

## Summary and outlook

We have demonstrated that treatment of obese rats or minipigs with a selective MC4-R peptide agonist MC4-NN1-0182 caused weight loss, which is associated with a decrease in food intake. Moreover, we have demonstrated both acute and subchronic weight-independent effects on insulin sensitivity. Overall, our observations identify MC4-R agonism as a viable target for the treatment of obesity and possibly also the insulin resistance which is a central factor involved in the pathogenesis of type 2 diabetes.

## Author contribution statement

All authors were involved in the design of the studies, K F, K R, and K D performed the studies. K F, K R, and B S W wrote the manuscript and C N and K D edited the manuscript.

## Figures and Tables

**Table 1:** Effect of MC4-NN1-0182 on plasma lipids and hormones and mesenteric fat depot in DIO rats. This table summarizes the level of plasma parameters including lipids and hormones as well as the size of the mesenteric fat depot at termination in DIO rats treated with MC4-NN1-0182 or vehicle, see Materials and methods for details; t-Chol, total cholesterol. Data are mean±s.e.m., *n*=10/group

**Measured parameters**	**Chow control**	**Vehicle**	**MC4-NN1-0182** (0.3 mg/kg)	**MC4-NN1-0182** (0.1 mg/kg)	**Pair-fed to MC4-NN1-0182** (0.3 mg/kg)
t-Chol (mM)	3.3±0.2	3.7±0.3	3.1±0.1^‡^	3.1±0.1^‡^	3.2±0.2
LDL-C (mM)	0.3±0.0*	0.7±0.1	0.6±0.0	0.5±0.0	0.5±0.1
HDL-C (mM)	1.8±0.1	2.0±0.1	1.6±0.1^†^	1.8±0.1	2.0±0.1
TAG (mM)	1.8±0.1	1.9±0.2	2.4±0.2	2.2±0.2	2.1±0.2
NEFA (μM)	53±13	99±15	137±15	104±7	133±25
Leptin (pM)	588±78	769±82	343±22^†^	495±106^‡^	403±21^†^
Insulin (pM)	551±61	553±101	354±34^‡^	360±49^‡^	426±54
Glucose (mM)	7.9±0.3	7.9±0.1	8.2±0.4	8.5±0.2	8.5±0.2
Mesenteric fat (g)	1.3±0.1	2.3±0.2	1.6±0.2^†^	1.5±0.2^†^	1.7±0.1^†^

Data were compared by one-way ANOVA followed by Bonferroni's multiple comparison test where *P<0.001, ^†^*P*<0.01, ^‡^*P*<0.05 vs vehicle control.

## References

[bib1] Andersen L, Dinesen B, Jørgensen PN, Poulsen F, Røder ME (1993). Enzyme immunoassay for intact human insulin in serum or plasma. Clinical Chemistry.

[bib2] Banno R, Arima H, Sato I, Hayashi M, Goto M, Sugimura Y, Murase T, Oiso Y (2004). The melanocortin agonist melanotan II increases insulin sensitivity in OLETF rats. Peptides.

[bib3] Berg F, Gustafson U, Andersson L (2006). The uncoupling protein 1 gene (*UCP1*) is disrupted in the pig lineage: a genetic explanation for poor thermoregulation in piglets. PLoS Genetics.

[bib4] Bertolini A, Gessa GL, Vergoni W, Ferrari W (1968). Induction of sexual excitement with intraventricular ACTH; permissive role of testosterone in the male rabbit. Life Sciences.

[bib5] Cannon B, Nedergaard J (2004). Brown adipose tissue: function and physiological significance. Physiological Reviews.

[bib6] Chen AS, Metzger JM, Trumbauer ME, Guan XM, Yu H, Frazier EG, Marsh DJ, Forrest MJ, Gopal-Truter S, Fisher J (2000). Role of the melanocortin-4 receptor in metabolic rate and food intake in mice. Transgenic Research.

[bib7] Clapham JC, Arch JR (2011). Targeting thermogenesis and related pathways in anti-obesity drug discovery. Pharmacology & Therapeutics.

[bib8] Conde-Frieboes K, Thøgersen H, Lau JF, Sensfuss U, Hansen TK, Christensen L, Spetzler J, Olsen HB, Nilsson C, Raun K (2012). Identification and *in vivo* and *in vitro* characterization of long acting and melanocortin 4 receptor (MC4-R) selective α-melanocyte-stimulating hormone (α-MSH) analogues. Journal of Medicinal Chemistry.

[bib9] Emmerson PJ, Fisher MJ, Yan LZ, Mayer JP (2007). Melanocortin-4 receptor agonists for the treatment of obesity. Current Topics in Medicinal Chemistry.

[bib10] Fan W, Boston BA, Kesterson RA, Hruby VJ, Cone RD (1997). Role of melanocortinergic neurons in feeding and the *agouti* obesity syndrome. Nature.

[bib11] Fan W, Dinulescu DM, Butler AA, Zhou J, Marks DL, Cone RD (2000). The central melanocortin system can directly regulate serum insulin levels. Endocrinology.

[bib38] Ferrannini E (1993). Syndrome X. Hormone Research.

[bib12] Finegood DT, Bergman RN, Vranic M (1987). Estimation of endogenous glucose production during hyperinsulinemic–euglycemic glucose clamps. Comparison of unlabeled and labeled exogenous glucose infusates. Diabetes.

[bib13] Fosgerau K, Fledelius C, Pedersen KE, Kristensen JB, Daugaard JR, Iglesias MA, Kraegen EW, Furler SM (2006). Oral administration of glucose promotes intracellular partitioning of fatty acid toward storage in white but not in red muscle. Journal of Endocrinology.

[bib14] Heijboer AC, van den Hoek AM, Pijl H, Voshol PJ, Havekes LM, Romijn JA, Corssmit EPM (2005). Intracerebroventricular administration of melanotan II increases insulin sensitivity of glucose disposal in mice. Diabetologia.

[bib15] Hill JW, Elias CF, Fukuda M, Williams KW, Berglund ED, Holland WL, Cho YR, Chuang JC, Xu Y, Choi M (2010). Direct insulin and leptin action on pro-opiomelanocortin neurons is required for normal glucose homeostasis and fertility. Cell Metabolism.

[bib16] Huszar D, Lynch CA, Fairchildhuntress V, Dunmore JH, Fang Q, Berkemeier LR, Gu W, Kesterson RA, Boston BA, Cone RD (1997). Targeted disruption of the melanocortin-4 receptor results in obesity in mice. Cell.

[bib17] Kievit P, Halem H, Marks DL, Dong JZ, Glavas MM, Sinnayah P, Pranger L, Cowley MA, Grove KL, Culler MD (2013). Chronic treatment with a melanocortin-4 receptor agonist causes weight loss, reduces insulin resistance, and improves cardiovascular function in diet-induced obese rhesus macaques. Diabetes.

[bib18] Kumar KG, Sutton GM, Dong JZ, Roubert P, Plas P, Halern HA, Culler MD, Yang H, Dixit VD, Butler AA (2009). Analysis of the therapeutic functions of novel melanocortin receptor agonists in MC3R-and MC4R-deficient C57BL/6J mice. Peptides.

[bib19] Lee M, Kim A, Chua SC, Obici S, Wardlaw SL (2007). Transgenic MSH overexpression attenuates the metabolic effects of a high-fat diet. American Journal of Physiology. Endocrinology and Metabolism.

[bib20] Lerner AB, McGuire JS (1961). Effect of α- and β-melanocyte stimulating hormones on the skin colour of man. Nature.

[bib21] Li G, Zhang Y, Wilsey JT, Scarpace PJ (2004). Unabated anorexic and enhanced thermogenic responses to melanotan II in diet-induced obese rats despite reduced melanocortin 3 and 4 receptor expression. Journal of Endocrinology.

[bib22] Li G, Zhang Y, Wilsey JT, Scarpace PJ (2005). Hypothalamic pro-opiomelanocortin gene delivery ameliorates obesity and glucose intolerance in aged rats. Diabetologia.

[bib23] MacNeil DJ, Howard AD, Guan XM, Fong TM, Nargund RP, Bednarek MA, Goulet MT, Weinberg DH, Strack AM, Marsh DJ (2002). The role of melanocortins in body weight regulation: opportunities for the treatment of obesity. European Journal of Pharmacology.

[bib24] Madsen AN, Hansen G, Paulsen SJ, Lykkegaard K, Tang-Christensen M, Hansen HS, Levin BE, Larsen PJ, Knudsen LB, Fosgerau K (2010). Long-term characterization of the diet-induced obese and diet-resistant rat model: a polygenetic rat model mimicking the human obesity syndrome. Journal of Endocrinology.

[bib25] Marsh DJ, Hollopeter G, Huszar D, Laufer R, Yagaloff KA, Fisher SL, Burn P, Palmiter RD (1999). Response of melanocortin-4 receptor-deficient mice to anorectic and orexigenic peptides. Nature Genetics.

[bib26] Matsuda M, DeFronzo RA (1999). Insulin sensitivity indices obtained from oral glucose tolerance testing: comparison with the euglycemic insulin clamp. Diabetes Care.

[bib27] Mul JD, van Boxtel R, Bergen DJM, Brans MAD, Brakkee JH, Toonen PW, Garner KM, Adan RAH, Cuppen E (2012). Melanocortin receptor 4 deficiency affects body weight regulation, grooming behavior, and substrate preference in the rat. Obesity.

[bib28] Nguyen N, Champion JK, Ponce J, Quebbemann B, Patterson E, Pham B, Raum W, Buchwald JN, Segato G, Favretti F (2012). A review of unmet needs in obesity management. Obesity Surgery.

[bib29] Obici S, Feng ZH, Tan JZ, Liu LS, Karkanias G, Rossetti L (2001). Central melanocortin receptors regulate insulin action. Journal of Clinical Investigation.

[bib30] Pierroz DD, Ziotopoulou M, Ungsunan L, Moschos S, Flier JS, Mantzoros CS (2002). Effects of acute and chronic administration of the melanocortin agonist MTII in mice with diet-induced obesity. Diabetes.

[bib39] Reaven GM (1998). Insulin resistance and human disease: a short history. Journal of Basic and Clinical Physiology and Pharmacology.

[bib31] Sebhat IK, Martin WJ, Ye ZX, Barakat K, Mosley RT, Johnston DBR, Bakshi R, Palucki B, Weinberg DH, Macneil T (2002). Design and pharmacology of *N*-[(3*R*)-1,2,3,4- tetrahydroisoquinolinium-3-ylcarbonyl]-(1*R*)-1-(4- chlorobenzyl)2-[4-cyclohexyl-4-(1*H*-1,2,4-triazol-1- ylmethyl)piperidin-1-yl]-2-oxoethylamine (1), a potent, selective, melanocortin subtype-4 receptor agonist. Journal of Medicinal Chemistry.

[bib32] Ujjainwalla F, Sebhat IK (2007). Small molecule ligands of the human melanocortin-4 receptor. Current Topics in Medicinal Chemistry.

[bib33] Vaisse C, Clement K, Guygrand B, Froguel P (1998). A frameshift mutation in human MC4R is associated with a dominant form of obesity. Nature Genetics.

[bib34] Vergoni AV, Poggioli R, Bertolini A (1986). Corticotropin inhibits food intake in rats. Neuropeptides.

[bib35] Wikberg JES, Muceniece R, Mandrika I, Prusis P, Lindblom J, Post C, Skottner A (2000). New aspects on the melanocortins and their receptors. Pharmacological Research.

[bib36] Yeo GS, Farooqi IS, Aminian S, Halsall DJ, Stanhope RC, Orahilly S (1998). A frameshift mutation in *MC4R* associated with dominantly inherited human obesity. Nature Genetics.

[bib37] Zhou L, Sutton GM, Rochford JJ, Semple RK, Lam DD, Oksanen LJ, Thornton-Jones ZD, Clifton PG, Yueh CY, Evans ML (2007). Serotonin 2C receptor agonists improve type 2 diabetes via melanocortin-4 receptor signaling pathways. Cell Metabolism.

